# A Portable Stiffness Measurement System

**DOI:** 10.3390/s17112686

**Published:** 2017-11-21

**Authors:** Onejae Sul, Eunsuk Choi, Seung-Beck Lee

**Affiliations:** 1 Institute of Nano Science and Technology, Hanyang University, 222 Wangsimni-ro, Seongdong-gu, Seoul 04763, Korea; ojsul@hanyang.ac.kr; 2 Department of Electronic Engineering, Hanyang University, 222 Wangsimni-ro, Seongdong-gu, Seoul 04763, Korea; silver77@hanyang.ac.kr

**Keywords:** stiffness measurement, Sneddon’s solution, portable system

## Abstract

A new stiffness measurement method is proposed that utilizes the lateral deformation profile of an object under indentation. The system consists of a force measurement module between a pair of equidistant touch sensing modules. Unique feature of the method is that by adjusting the touch module separation, indenter protrusion, and spring constant of the force sensing module, one can choose a desired sensing range for the force module. This feature helps to enhance the stiffness differentiation between objects of similar hardness and avoids measurement saturation. We devised a portable measurement system based on the method, and tested its performance with several materials including polymer foams and human skin.

## 1. Introduction

Stiffness is an important factor that is needed to perceive the properties of an object through tactile interaction. Intuitively, the stiffness can be understood as the responsive deformation of an object’s surface under an indentation force. Thus one only needs to know the deformation depth and the magnitude of the force applied to determine its stiffness. However, it is difficult to measure the deformation depth of the contacting surface directly while at the same time measuring the applied force. One way to evade this difficulty is to use a cantilever. When the tip of a cantilever is pressed onto an object's surface, the deflection of the cantilever will indicate the indentation depth of the object surface and by pre-measured force-dependent deflection data the amount of applied force may be known allowing the stiffness to be measured [1,2]. Although highly sensitive, the cantilever structure would not be mechanically stable limiting the sensor application to relatively soft objects only. There are additional stiffness measurement methods that do not require measurement of the deformation depth, such as methods that use two springs having differing spring constants, and another that use resonant vibrational frequencies [3,4,5,6,7,8,9,10,11,12]. When two springs are supported by a common frame and they are pressed against an object, the compression of each spring will be different, if the spring constants are different. Then the elastic modulus of the contacting object will be related to the relative compression of the springs. There have been many reports of stiffness measurements using this principle [3,4,5,6] with all of the systems requiring simultaneous measurements of two or more pressure sensors, or piezoelectric films [7] in parallel which, for soft objects, may lead to fluctuation and noise in the measured stiffness, reducing accuracy. When a pressurized mechanical vibration is applied to a contact surface, there will be a resonant vibrational frequency that depends of the acoustic impedance of the contacting surface. The shift in resonant frequency or the shift in phase of the recovery response due to repetitive indentation will depend on the stiffness of the object allowing its measurement [8,9,10,11,12]. Although highly sensitive, this type of system requires mechanical feedback from the object to create measureable shifts in vibrational frequencies limiting sensitivity to relatively soft materials.

Here, we introduce a portable stiffness measurement system that does not require pressure dependent indentation depth measurement to determine the stiffness of the contacted surface. Stiffness, *k*, is defined as the magnitude of a force, *F*, acting on an object, divided by a deformation, *δ*, of the object (*k* = *F*/*δ*). Our measurement method utilizes the fact that for the same deformation normal to the force, a different amount of force would be required for surfaces with differing stiffness.

**Figure 1:**
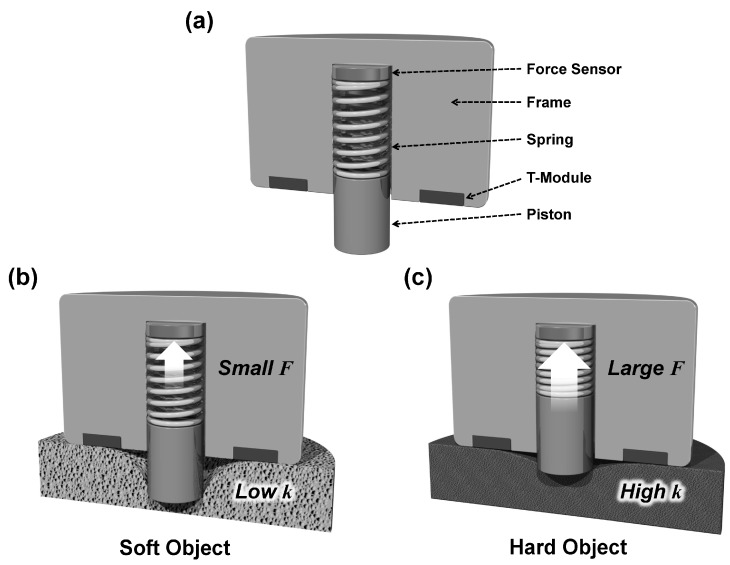
Schematic illustration of the stiffness measurement system. (**a**) Cross-sectional schematic diagram of the initial state of the stiffness measurement system. Comparison between measurements of (**b**) soft and (**c**) hard objects.

Figure 1 illustrates the operating principle of our stiffness measurement system. The system consists of a force module (f-module), a cylindrical piston attached to the frame of the system through a spring with a spring constant *k*_1_ and a force sensor, and a pair of touch modules (t-module), a contact detection sensor. To measure the stiffness of an object, we make contact to the object by the piston portion of the f-module. Here we assume that the object has a flat surface with no roughness. Then force is applied via the piston until the t-module detects contact (Figure 1a). If the object is relatively soft, it will require less force before the object makes contact with the t-module and the compression of the spring will be small giving a relatively low stiffness value (Figure 1b). While for a harder object, it will require higher forces for the object to make contact with the t-module and the compression of the spring will be large giving a relatively high stiffness value (Figure 1c). Therefore, by simply pressing the piston on the object until the t-module makes contact and recording the measured force applied at that instant, it will be possible to measure the stiffness of the contacting surface. As it will be shown, the system can be specified to measure various ranges of stiffness and the operating principle allows the system to be scalable making it possible for the sensing module to be integrated into various passive instruments giving them the ability to sense the stiffness of objects under manipulation.

## 2. Operating Principle of the Stiffness Measurement System

**Figure 2:**
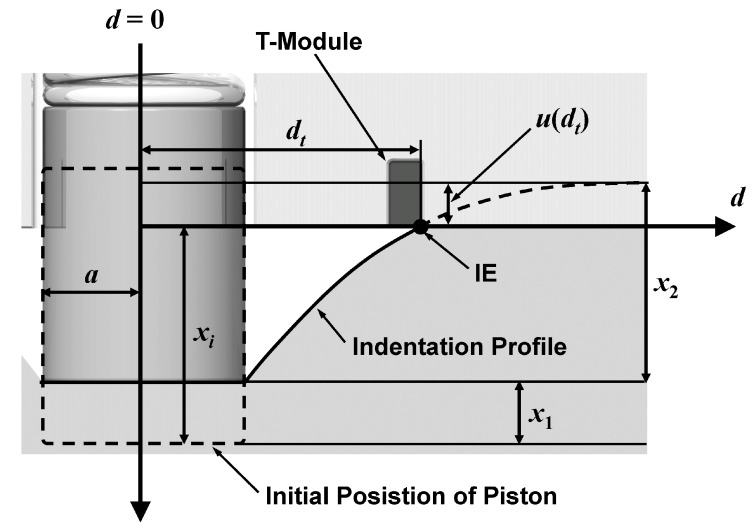
Cross-section diagram of the system during pressurizing stiffness measurement.

The cross-section of the plane being pressurized will have an indentation profile following that from the Sneddon’s equation (Equation (3)) [13], which will depend on the pressure and the object’s stiffness (Figure 2). A distance, *d* is defined as the distance from the center of the piston to the indentation edge (IE, in short). When force is applied, the piston transfers the reactive force of the indented object to the force sensor inside the f-module. The t-module, whose edge is separated by a distance *d_t_* from the piston will determine the moment when the object makes contact to it and when to measure the force at the f-module. When the stiffness of the object increases, the indentation profile will have a lower curvature, requiring higher forces, wider *d_t_*, shallower piston, or lower spring constant (*k*_1_) to activate the t-module. Thus from a geometrical perspective, the stiffness measurement problem transforms to a relation between the location of the t-module and the range of the IE movement, according to the applied force.

The stiffness of the object with an effective spring constant *k*_2_ can be determined from the equations below. Let us assume that a piston with radius *a* has a spring constant *k*_1_, and it is indented by the object by a depth of *x*_1_. The forces experienced by the spring and the object are identical:

(1) $$F=k_{1}\cdot x_{1}=k_{2}\cdot x_{2}$$

The indented depth of the object by the piston is *x*_2_. At a specific force, *F*, the IE reaches the t-module, which is located a distance *d_t_* from the piston. At that moment, we have a relationship:

(2) $$x_{i}=x_{1}+x_{2}-u(d_{t})$$

Here a function, *u*(*d*) is defined as a vertical distance from the imaginary non-deformed surface of the object to the indentation profile at location *d*. When the IE is at *d_t_*, then *u* becomes *u*(*d_t_*). According to the Sneddon’s equation [13]:

(3) $$u(d_{t})=\frac{2x_{2}}{\pi }\arcsin(\frac{a}{d_{t}}).$$

Equation (3) is based on an assumption that the thickness of the object is infinite. If the indentation depth is much smaller than the thickness of the object, we can still use the equation without significant error. In our tests, the indentation depths were only 3~6 mm, but the thickness of our objects were more than 2 cm. Combining Equations (1)–(3), we have:

(4) $$k_{2}=\frac{1-\frac{2}{\pi }\arcsin(\frac{a}{d_{t}})}{\frac{x_{i}}{F}-\frac{1}{k_{1}}}$$

Thus stiffness *k*_2_ depends on four factors; the initial piston protrusion height *x_i_*, the spring constant *k*_1_, the separation distance *d_t_*, and the measured force *F* when the t-modules were activated.

## 3. Fabrication of the Stiffness Measurement System

**Figure 3:**
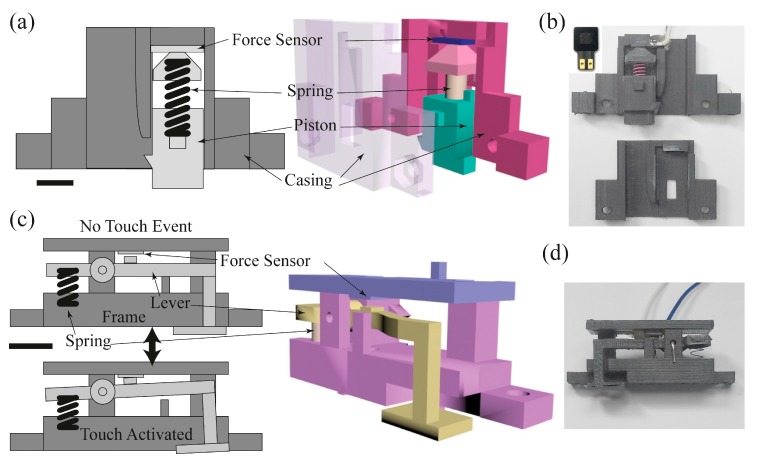
Design and assembly of the stiffness measurement system. (**a**) Cross-sectional schematic of the f-module, and (**b**) a picture of fabricated f-module parts. Inset shows a picture of the force sensor. (**c**) Cross-sectional schematic of the t-module, and (**d**) a picture of an assembled t-module. The scale bars in (**a**,**c**) represents 1 cm.

The stiffness measurement system consists of one f-module and two t-modules. It should be noted that the modules were designed to produce a system that will effectively demonstrate the proposed stiffness measuring principle and not optimized for a particular sensing range or reliability. The schematic cross-section diagrams of the f-module and the t-module are shown in Figure 3a,c. For the f-module, we used a commercially available force sensor (QA6P, Marveldex, Bucheon, Korea) and a spring (SWC6-20, *k* = 2.7 N/mm, Misumi Spring, Schaumburg, IL, USA). The polymer casing that houses the f-module was fabricated using a 3D printer (Figure 3b). For the t-module, we used the identical force sensor, but we devised a lever structure to reduce the force threshold to detect a much lighter contact force compared to the force applied to the f-module. (Figure 3c,d) When a force is applied at the foot of the lever, a small tab in the middle of the lever presses the force sensor. When the force is removed, the lever foot returns to its original location by a spring (WY5-15, *k* = 0.1 N/mm, Misumi Spring). Two t-modules were fabricated with a mirrored structure necessary for balance and symmetry. The structures holding the lever, spring, and the force sensor were also 3D-printed. Any other type of sensor can be used without the lever for the t-module, if they have a small enough force threshold for touch detection. Also, without the lever mechanism, a smaller module design can be possible, minimizing the whole footprint of the stiffness measurement system.

## 4. Calibration of f-Module and t-Modules

**Figure 4:**
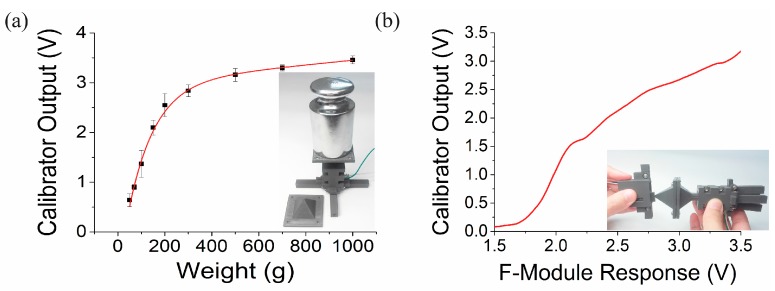
F-module sensor calibration. (**a**) Weight dependent output of the calibration tool. Its transposed curve was fit to a polynomial equation. (See Supplementary Materials.) Inset shows a picture of the measurement process. (**b**) Calibration of the f-module output voltage. The curve was fit to a polynomial equation in the Supplementary Materials. The bias of the f-module response came from the pre-load of the spring in the f-module. Inset shows a picture showing the application of force to the f-module by the calibration tool using the pyramidal tip in (**a**).

The f-module has a force sensor connected to a piston (Figure 3a). When the module is pressed on an object, the reactive force from the object is applied to the piston, and the force is transferred to the sensor. The magnitude of the force is recorded by the resistance change of the force sensor. The f-module could be calibrated in principle by putting a weight on the piston, but the area of the piston cross-section is too narrow for weights. Thus we devised a calibration tool accommodating heavy weights up to 1 kg (inset of Figure 4a). The working principle and internal structure for measuring the force was identical to the one in the Figure 3a except that it has a much wider piston. Figure 4a shows the measurement result of the calibration tool. There were nine different weights from 70 g to 1 kg applied on the tool, and the force sensor output voltage was linear up to 200 g and then began to saturate as weights were increased further. Next, the calibration tool was used to calibrate the f-module by pressing the piston of the f-module against the piston of the calibration tool. Application of force was conducted several times and the output voltages were recorded, averaged, and fitted to a conversion curve as shown in Figure 4b. We believe the small steps visible in the data were due to some friction between the 3D printed parts and were not a characteristic of the force sensors. Thus it was now possible to know how much force was applied by reading the force sensor output of the f-module. Then the force was inserted in Equation (4) to obtain the stiffness.

**Figure 5:**
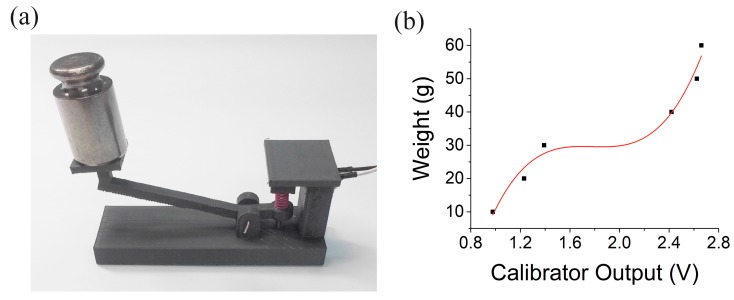
T-module sensor calibration. (**a**) Image of the t-module calibration tool. (**b**) Response curve from the calibration tool.

For the t-modules, the threshold force should be minimized since it should be possible to activate them with just a very weak contact. To do so, we used a lever mechanism (Figure 3c). Unlike the f-module, the t-module does not need to respond to a wide range of forces. Instead, it requires reliable activation at the same minimal amount of applied force. Because the t-modules also have a narrow contact area, we also devised a separate calibration tool for the t-module as shown in Figure 5a. Using various weights from 10 g to 60 g, we obtained a response curve from the tool (Figure 5b). Similarly to the method shown in the inset of Figure 4b, the t-module was calibrated. The foot of the t-module lever was pressed against the t-module calibrator, and the output of the calibrator was converted to force using the curve in Figure 5b. The threshold force was 26 ± 3 g for ten t-modules we tested.

## 5. Measurement of Stiffness

### 5.1. Stiffness Measurement Module Assembly

**Figure 6:**
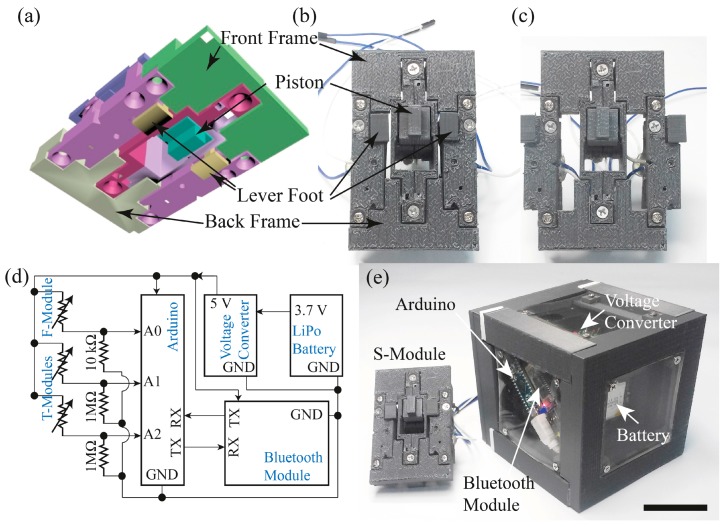
Assembly of the portable stiffness measurement system. (**a**) 3-dimensional schematic illustration of the assembled s-module. (**b**) A picture of the assembled s-module, and (**c**) the same module with interchanged t-module placements. (**d**) A circuit diagram of the stiffness measurement system. (**e**) A picture of the completed system. The scale bar represents 5 cm.

The f-module and two t-modules were assembled into the stiffness sensing module (s-module). The f and t-modules were held by two frames; the front frame and the back frame (Figure 6a,b). The separation of t-modules from the f-module *d_t_*, can be adjusted by interchanging the locations of the two t-modules as shown in Figure 6c. The stiffness measuring system was designed to be portable with a dedicated control circuitry, communication module and a power supply, all assembled into an independent system. A driving circuit was prepared as shown in Figure 6d. The inputs from the f-module and t-modules were connected to the analog inputs of a micro-controller board (Arduino Uno, Arduino, Turin, Italy). For the f-module, a 5 V bias was provided by the Arduino board, and the sensor was serially connected to a 10 kΩ resistor. The output of the sensor was fed into an analog input by a pull-down method. When no force was applied to the sensor, the sensor was basically an open switch, and the input terminal will record 0 V. When there was a force above threshold, the sensor passes a current and the input will record a finite voltage below 5 V. The wiring for the t-module was identical to that of the f-module, a pull-down connection to an analog input to the Arduino board with a 1 MΩ resistor. The activation of the t-module was identified when the input voltage was above a specified voltage to the input channel. Power was supplied by a Lithium-polymer (LiPo in short) battery which produces 3.7 V. The voltage was shifted up to 5 V through a voltage shifter (PowerBoost 1000, Adafruit Industries, New York, NY, USA), and it was supplied to the Arduino and a Bluetooth module (HC-06, Waveshare, Shenzhen, China). A Bluetooth module was connected to the Arduino board for wirelessly transferring the measured voltages to a computer. Figure 6e shows a picture of the completed system.

**Figure 7:**
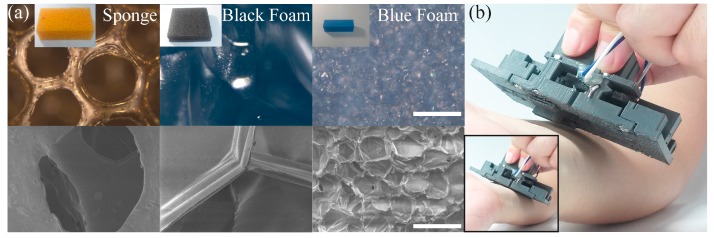
Demonstration of stiffness measurement. (**a**) Various objects for stiffness measurements. Top row: optical images taken at 100 times magnitude. The scale bar represents 200 μm. Insets: pictures of the objects. The dimension of sponge, black foam, and blue foam were 9 cm (length) × 5 cm (width) × 2.5 cm (thickness), 12 cm × 10 cm × 2 cm, and 8 cm × 3 cm × 3 cm, respectively. Bottom row: scanning electron microscope images of the same materials in the top row taken at 200 times magnitude. The scale bar represents 100 μm. (**b**) A picture showing a measurement being taken on the forearm skin. Inset shows the moment when the t-modules were activated.

Four materials with different stiffness were chosen for stiffness measurement demonstration; sponge made of polyurethane, black foam made of polystyrene, blue foam made of polyolefin (Figure 7a), and human forearm skin. For measurements, the top portion of the f-module was hand-held, and the s-module was pressed against the object until both of the t-modules were activated, as shown in Figure 7b. The moment of activation was registered by an Arduino code, and the force at the moment was recorded. Ten repeated measurements were done per object with the t-module activation set at 26 ± 3 g measured force.

In Section 2, Equation (3) shows that the measurement depends on three factors, *d_t_*, *k*_1_, and *x_i_*. In the following subsections, we will show how the stiffness discriminating capability of the s-module was modified, by adjusting piston protrusion, spring constant, and the t-module separation distance.

### 5.2. Effect of t-Module Separation Length Difference

**Figure 8:**
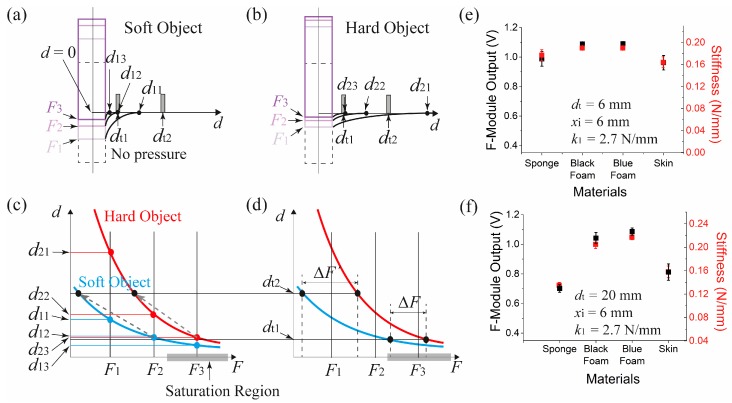
T-module separation dependent stiffness measurement. Illustration of the variation of the indentation profile and IE in case of a softer object (**a**), and (**b**) in case of a harder object. (**c**) Distribution of the IE position depending on applied force. (**d**) t-module position dependent measured forces showing sensing range selection based on t-module separation. Stiffness measurement obtained when the separation was (**e**) 6 mm, and (**f**) 20 mm. For both cases, *k*_1_ was 2.7 N/mm, and *x_i_* was 6 mm.

When force is applied to an object through the piston, the object surface will start to deform accordingly. With increased force, the object surface will touch the s-module bottom surface and the edge of the surface indentation IE point will be definable. The IE will approach toward the center as the reactive force received by the object increases. For a softer object, the indentation depth will be relatively deep and the IE movement range will be narrow and close to the piston (Figure 8a). For a harder object, the indentation depth will be relatively shallow, and the IE movement range will be relatively wider and farther from the piston since the surface will be less deformable (Figure 8b). When the force is *F*_1_, the IE of the two cases will be *d*_11_ and *d*_21_ each. In both cases, the t-modules located at distance *d_t_*_1_ will not be activated and no measurement will be recorded. If the softer object is pressed further until the force becomes *F*_2_, at which point *d*_12_ = *d_t_*_1_, then the t-module will be activated and the stiffness will be recorded. However at the same force with the harder object, the t-module will not be activated still (*d*_22_ > *d_t_*_1_). The harder object needs to be pressed further until *F*_3_ is applied to activate the t-module (*d*_23_ = *d_t_*_1_). Thus the difference of the recorded force indicates the difference of the stiffness between the two objects.

In principle, the location of the touch module should not matter for the measurement, but usually a force sensor has a saturation range at high pressures. In that case, the recorded force values, for example, *F*_2_ and *F*_3_ will not be much different in terms of the sensor output (see Figure 4a). To avoid this problem, one can use the more linear range of the sensor simply by locating the t-modules further away, from *d_t_*_1_ to *d_t_*_2_. In this new arrangement, the harder object’s stiffness will be recorded at a force less than *F*_2_, instead of *F*_3_, and the softer object will touch the t-module at a force less than *F*_1_ (dashed arrows and black dots in Figure 8c). Thus our system has the advantage that by adjusting the t-module separation it will be possible to choose the sensing region of the force sensor for better stiffness discrimination. Note that the recorded force difference *ΔF’* at *d_t_*_2_, becomes larger than *ΔF* at *d_t_*_1_ and the new measurements avoids the saturation range of the force sensor (a numerical plot of Figure 8d is shown in Figure S1 of the Supplementary Materials).

Figure 8e,f show the effect before and after the separation distance change. It can be seen that the sensor outputs were not discernible between the objects when the separation was 6 mm, but after the separation was increased to 20 mm, the stiffness difference became obvious. For measuring extremely soft surfaces with very low surface tension, it may be desirable to have the t-module separation be reduced making the force sensor operate above its threshold for better force measurement reliability. The measured voltages in Figure 8f were converted to the stiffness via module calibration and Equation (4). The stiffness of the tested objects were between 0.13 N/mm and 0.22 N/mm. To verify these results, we measured stiffness separately by recording deformation of the objects under known weights. As a result, we obtained 0.098 N/mm (30 kPa) 0.227 N/mm (57 kPa), and 0.250 N/mm (94 kPa) for the sponge, the black foam, and the blue foam, respectively. Also, these numbers were similar in range to the estimated stiffness (0.1–1 N/mm) of the materials based on their Young’s moduli [14,15,16] and of their geometries showing that the proposed stiffness measurement method was applicable. Stiffness of the skin cannot be obtained by this method. But, according to [17], the Young’s modulus of skin is about 10–20 kPa. If we assume that the skin thickness was roughly 1 cm and the area of skin under indentation was 0.8 cm^2^ (0.55 cm × 1.45 cm), the stiffness of the skin can be calculated using the following equation:

(5) $$k=\frac{EA}{t}=\frac{10\sim 20\;\mathrm{kPa}\times 0.8\;{\mathrm{cm}}^{2}}{10\;\mathrm{mm}}=0.08\sim 0.16\;{N/\mathrm{mm}}^{2}$$

where *k* is the stiffness, *E* is the Young’s modulus, *A* is the indentation area, and *t* is the thickness. The calculated stiffness of skin is 0.08~0.16 N/mm^2^, which roughly agrees with our result. Further calibration and system optimization should be able to increase measurement accuracies.

### 5.3. Effect of Piston Protrusion Difference

In the previous subsection, we have shown that the t-module separation can enhance the stiffness discrimination ability. But for some applications, the t-module separation can be limited due to geometrical considerations. Also requirement on local stiffness assessment can limit the size of the s-module itself. In these cases, large separation will not be possible, and other alternative methods for enhancing the discrimination ability should be investigated. One method is to change the piston protrusion (*x_i_*), and the other is to change the spring constant (*k*_1_).

**Figure 9:**
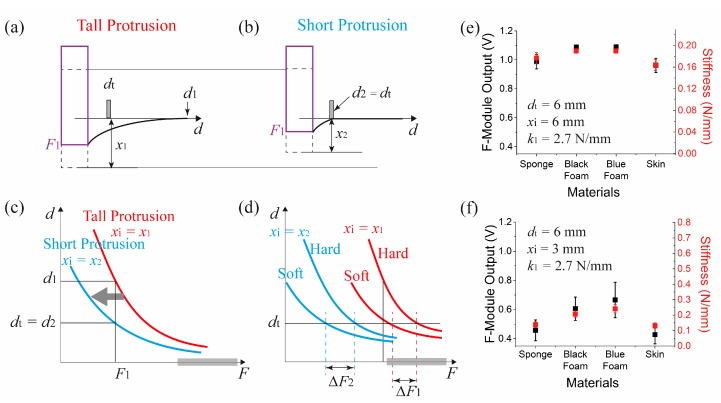
Piston protrusion dependent stiffness measurement. Illustration of the variation of IE of a soft object for (**a**) a tall protrusion and (**b**) a short protrusion. (**c**) Distribution of the IE position depending on applied force. (**d**) Piston protrusion dependent force sensing range selection. Stiffness measurement obtained when the piston protrusion (*xi*) was (**e**) 6 mm and (**f**) 3 mm. For both cases, *d_t_* was 6 mm, and *k*_1_ was 2.7 N/mm. (note that (**e**) is the same as Figure 8e).

Figure 9a–d show the effect of piston protrusion on the measurements. Let us assume that there are two identical s-modules with the same *d_t_* and same *k*_1_, except different piston protrusions from the bottom of the s-module. When two identical objects are pressed against them with identical force *F*_1_, the pistons indentation will be the same, but their IE points will be different. At this force, the IE for the short protrusion *d*_2_ will coincide with the t-module position at *d_t_* (=*d*_2_), while the IE for the tall protrusion *d*_1_ will be larger than *d*_2_. Thus compared to the tall protrusion module, one can apply smaller forces for stiffness measurement. For shorter protrusions, the result will be a shift of the IE point curve in Figure 9c toward the left and reduction of the force required to measure the stiffness. This feature is advantageous when there are two objects consisting of relatively soft and hard materials that require smaller t-module separation due to their size limitation. If their measurements are recorded near the saturation region of the force sensor, one can reduce the protrusion of the piston to shift the measurement range to below the sensor saturation region (Figure 9d).

The effect of protrusion change was verified from the two data sets shown in the Figure 9e,f. They show two sets of measurements when the protrusion of the piston was 6 mm and 3 mm each. When the 3 mm protrusion was used, the measured force difference between objects became enlarged enough so that the stiffness of soft materials like sponge or skin were distinct from that of the foams.

### 5.4. Effect of Spring Constant Difference

**Figure 10:**
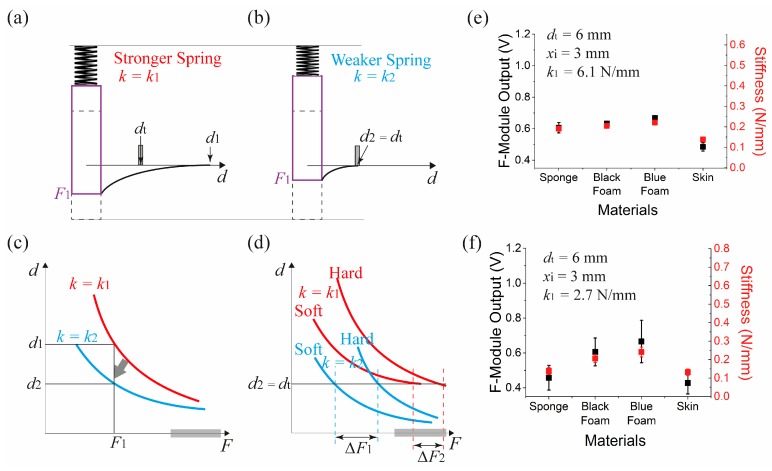
Spring constant dependent stiffness measurement. Illustration of the variation of IE for a stronger spring (**a**) and a weaker spring (**b**). (**c**) Distribution of the IE position depending on the spring constant. (**d**) Spring constant dependent force sensing range selection. Stiffness measurement obtained when the spring constant was (**e**) 6.1 N/mm and (**f**) 2.7 N/mm. For both cases, *d_t_* was 6 mm, and *x_i_* was 3 mm. (Note, (**f**) is the same as Figure 9f).

Figure 10 shows the effect of spring constant difference on the stiffness measurement. Figure 10a,b compare the difference of IE locations under identical applied force, *F*_1_. In case of the relatively stronger spring, *d*_1_ does not reach *d_t_* and no force is measured (Figure 10a). For the weaker spring, the piston is indented further resulting in a closer IE and *d*_2_ reaches *d_t_*, and a measurement of the applied force is made. Thus the effect of changing the spring constant is to shift the IE dependence on the applied force, as shown in Figure 10c. For a fixed t-module position at *d*_2_ (=*d_t_*), we can see from Figure 10d, that using the softer spring results in a wider separation in the measured forces for the soft and hard objects because it was possible to avoid the force sensor saturation region. This was demonstrated experimentally by comparing two datasets shown in Figure 10e,f. 

Here the two s-modules have identical configurations including the t-module separation (*d_t_* = 6 mm) and spring protrusion (*x_i_* = 3 mm), except for the spring constant. For the case of the weaker spring (*k*_1_ = 2.7 N/mm) (Figure 10f), the dispersion of the force measurements were clearer than that of the case using the stronger spring (*k*_1_ = 6.1 N/mm: SWC10-15, Misumi Spring) (Figure 10e).

We can understand the dependence on the spring constant by considering the deformation of contacting objects. For the stronger spring, an object needs to be pressed harder for the stiffness measurement to take place. It results in higher pressure applied to the force sensor and the measurement to be taken near the saturation region of the sensor.

From the results presented, we find that our method of stiffness measurement overcomes the need for depth measurement, or simultaneous measurement of several force sensors in parallel. Also by alternating passive elements, our stiffness measuring system can be adjusted to measure stiffness at various ranges for better discrimination of stiffness between different objects. Moreover, the operating principle is simple enough that the sensor components may be scaled and optimized to fit onto much smaller areas allowing it to be integrated into passive manipulation tools used today in such applications as, medical examination, remote surgery, and artificial robotic fingers.

## 6. Conclusions

We have introduced a stiffness measurement system that utilizes the contacting object’s indentation profile depending on applied force. The indentation edge is the key parameter for determination of the stiffness measurement, because it is the triggering factor for the measurement of the stiffness of an object. To enhance the discrimination capability of the system, sensitivity of the IE movement depending on pressure could be controlled by adjusting three design parameters, the t-module separation, the piston protrusion, and the spring constant. Our stiffness measurement system may be scaled and optimized to be integrated into various tools and probes due to the simplicity in operation and the ability to adjust the sensing range to increase the stiffness measurement accuracy.

## Supplementary Materials

The following are available online at http://www.mdpi.com/1424-8220/17/11/2686/s1. Figure S1. Theoretical distribution of the IE position depending on applied force.
