# Supplementary material for: A Portable Stiffness Measurement System
Source: Sensors (Basel). 2017 Nov 21;17(11):2686. doi: 10.3390/s17112686 (PMC5712800; doi:10.3390/s17112686)
Supplement: Supplementary file 1 [file sensors-17-02686-s001.pdf]

Supplementary Material

A Portable Stiffness Measurement System

Onejae Sul [1], Eunsuk Choi [2] and Seung-Beck Lee [1,2,*]

[1] Institute of Nano Science and Technology, Hanyang University, 222 Wangsimni-ro, Seongdong-gu, Seoul 04763, Korea; ojsul@hanyang.ac.kr

[2] Department of Electronic Engineering, Hanyang University, 222 Wangsimni-ro, Seongdong-gu, Seoul 04763, Korea; silver77@hanyang.ac.kr

[*] Correspondence: sbl22@hanyang.ac.kr; Tel.: +82-2-2220-1676; Fax: +82-2-2294-1676

Fit equation from the transposed curve in Figure 4(a):

$$y = 18.753x^6 - 183.5918x^5 + 73.22399x^4 - 1502.83x^3 + 1627.3419x^2 - 803.6x + 187.052$$

Here, *y* is the force in grams, and *x* is the f-module calibrator output in voltage. The dependent variable is set to force and independent variable is set to calibrator output, which are switched from the *x* and *y*-axes in Figure 4(a). This equation was used starting from f-module output to calculate the force applied on the f-module, in conjunction with the equation below.
Fit equation from Figure 4(b):

$$y = 0.00547116x^9 - 0.0358632x^8 + 0.00611468x^7 + 0.287445x^6 - 0.204121x^5 - 0.891349x^4 + 0.477689x^3 + 1.49812x^2 + 0.5555x - 0.00196643$$

Here, *y* is the f-module calibrator output in voltage, and *x* is the f-module output in voltage.
Fit equation from Figure 5(b):

$$y = 41.0896x^3 - 219.40212x^2 + 389.66652x - 200.59051$$

Here, *y* is the force in grams, and *x* is the t-module calibrator output in voltage.

Relationship between the IE Position and the Contact Force

The relationship between IE position d_t and contact force F can be obtained from Equation (4):

$$d_t = \frac{a}{\sin\left(\frac{\pi}{2}\left(1 - k_2\left(\frac{x_i}{F} - \frac{1}{k_1}\right)\right)\right)}$$

This equation is plotted in Figure S1. Considering the dimension of our system, the parameters of a, x_i, and k_1 are set as 3 mm, 6 mm, 2.7 N/mm^2. The stiffness of soft and hard contact objects are assumed as 4 and 5 N/mm^2, respectively. 0.79 N of contact force difference at 10 mm of IE position, becomes larger than 0.82 N at 20 mm. Thus better stiffness discrimination can be obtained by adjusting the t-module separation length.

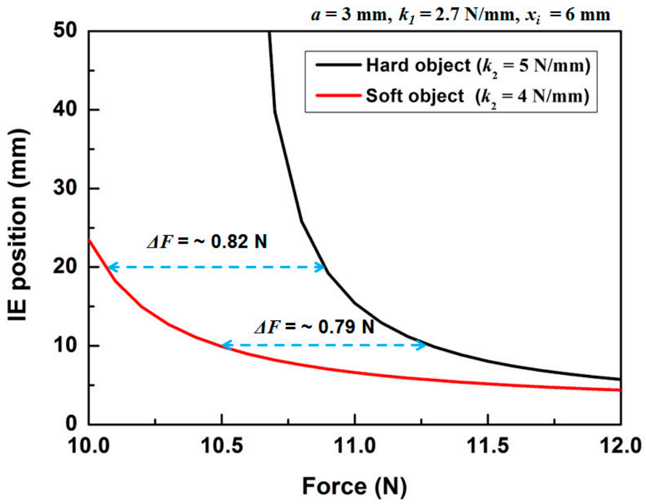

Figure S1. Theoretical distribution of the IE position depending on applied force.
